# Contact Challenge of Cattle with Foot-and-Mouth Disease Virus Validates the Role of the Nasopharyngeal Epithelium as the Site of Primary and Persistent Infection

**DOI:** 10.1128/mSphere.00493-18

**Published:** 2018-12-12

**Authors:** Carolina Stenfeldt, Ethan J. Hartwig, George R. Smoliga, Rachel Palinski, Ediane B. Silva, Miranda R. Bertram, Ian H. Fish, Steven J. Pauszek, Jonathan Arzt

**Affiliations:** a Foreign Animal Disease Research Unit, USDA-ARS, Plum Island Animal Disease Center, Greenport, New York, USA; b Department of Veterinary Population Medicine, University of Minnesota, St. Paul, Minnesota, USA; c Department of Diagnostic Medicine/Pathobiology, Kansas State University, Manhattan, Kansas, USA; d PIADC Research Participation Program, Oak Ridge Institute for Science and Education, Oak Ridge, Tennessee, USA; University of Pittsburgh School of Medicine

**Keywords:** foot-and-mouth disease, foot-and-mouth disease virus, FMD, FMDV, cattle, pigs, virus, pathogenesis, NGS, transmission

## Abstract

The pathogenesis of foot-and-mouth disease virus (FMDV) in cattle was investigated through early and late stages of infection by use of an optimized experimental model for controlled contact exposure. Time-limited exposure of cattle to FMDV-infected pigs led to primary FMDV infection of the nasopharyngeal mucosa in both vaccinated and nonvaccinated cattle. In nonvaccinated cattle, the infection generalized rapidly to cause clinical disease, without apparent virus amplification in the lungs prior to establishment of viremia. Vaccinated cattle were protected against clinical disease and viremia; however, all vaccinated cattle were subclinically infected, and persistent infection occurred at similarly high prevalences in both animal cohorts. Infection dynamics in cattle were consistent and synchronous and comparable to those of simulated natural and needle inoculation systems. However, the current experimental model utilizes a natural route of virus exposure and is therefore superior for investigations of disease pathogenesis and host response. Deep sequencing of viruses obtained during early infection of pigs and cattle indicated that virus populations sampled from sites of primary infection were markedly more diverse than viruses from vesicular lesions of cattle, suggesting the occurrence of substantial bottlenecks associated with vesicle formation. These data expand previous knowledge of FMDV pathogenesis in cattle and provide novel insights for validation of inoculation models of bovine FMD studies.

**IMPORTANCE** Foot-and-mouth disease virus (FMDV) is an important livestock pathogen that is often described as the greatest constraint to global trade in animal products. The present study utilized a standardized pig-to-cow contact exposure model to demonstrate that FMDV infection of cattle initiates in the nasopharyngeal mucosa following natural virus exposure. Furthermore, this work confirmed the role of the bovine nasopharyngeal mucosa as the site of persistent FMDV infection in vaccinated and nonvaccinated cattle. The critical output of this study validates previous studies that have used simulated natural inoculation models to characterize FMDV pathogenesis in cattle and emphasizes the importance of continued research of the unique virus-host interactions that occur within the bovine nasopharynx. Specifically, vaccines and biotherapeutic countermeasures designed to prevent nasopharyngeal infection of vaccinated animals could contribute to substantially improved control of FMDV.

## Abstract (precis)

Foot-and-mouth disease virus (FMDV) is an important livestock pathogen that is often described as the greatest constraint to global trade in animal products. The present study utilized a standardized pig-to-cow contact exposure model to demonstrate that FMDV infection of cattle initiates in the nasopharyngeal mucosa following natural virus exposure. Furthermore, this work confirmed the role of the bovine nasopharyngeal mucosa as the site of persistent FMDV infection in vaccinated and nonvaccinated cattle. The critical output of this study validates previous studies that have used simulated natural inoculation models to characterize FMDV pathogenesis in cattle and emphasizes the importance of continued research of the unique virus-host interactions that occur within the bovine nasopharynx. Specifically, vaccines and biotherapeutic countermeasures designed to prevent nasopharyngeal infection of vaccinated animals could contribute to substantially improved control of FMDV.

## INTRODUCTION

Foot-and-mouth disease (FMD), caused by FMD virus (FMDV; genus Aphthovirus, family Picornaviridae), is a contagious livestock disease of global concern (1). FMD affects cloven-hoofed domestic and wild animal species, including cattle, pigs, and small ruminants, and substantially impacts industrialized as well as subsistence farming systems in affected regions (2). Countries in which FMD is endemic are burdened by the costs and logistic challenges of FMD control measures, such as disease surveillance, repeated vaccination campaigns, and enforcement of zoosanitary measures for outbreak control. Additionally, trade restrictions enforced to prevent introduction of FMDV into countries that are currently free of the disease prevent access to international markets for trade in animal products for countries that have not managed to efficiently control FMD.

The clinical signs of FMD include fever, lameness, and salivation (ptyalism) associated with the appearance of characteristic vesicular lesions in the oral cavity and in the interdigital clefts and coronary bands of the feet (3, 4). Mortality rates are generally low among adult animals. However, important production objectives such as weight gain, milk yield, and draught power may be severely affected by the clinical disease.

FMDV is infectious in low doses and can be transmitted by both direct and indirect mechanisms, including movement of infected animals as well as mechanical transfer by contaminated vehicles and fomites. Cattle are specifically sensitive to FMDV infection via the respiratory route (4, 5). Early works localized the site of primary FMDV infection in cattle to the pharynx (6 – 8). More recent investigations have refined this knowledge by demonstrating that initial FMDV infection occurs within distinct regions of specialized epithelium associated with mucosa-associated lymphoid tissue (MALT) within the nasopharyngeal mucosa (9, 10). Additionally, it has been demonstrated that persistent FMDV infection, which occurs in a large proportion of both vaccinated and naive cattle, is localized to the same microanatomic regions of the nasopharyngeal mucosa (11, 12). Several investigations have shown that substantial FMDV replication may occur within the bovine lungs during the early phase of infection (9, 13 – 15). However, recent findings have suggested that the importance of the lungs as a site of FMDV amplification during early infection varies, depending on the route of virus exposure. Specifically, whereas FMDV exposure of cattle via controlled aerosol delivery resulted in substantial viral replication in the lungs (9, 16), there was no detection of virus amplification in the lungs before the onset of viremia in animals that had been infected through intranasopharyngeal (INP) deposition of FMDV (10).

Historically, challenge of cattle during experimental FMDV studies has been done through intraepithelial injection on the dorsal surface of the tongue (17). This inoculation system, generally (and erroneously) referred to as “intradermal lingual” (IDL) injection, also represents the FMDV exposure system recommended for FMDV vaccine testing by the World Organisation for Animal Health (OIE) (18). Tongue inoculation represents a consistent and stringent model for FMDV challenge studies. However, as tongue inoculation represents an unnatural route of virus entry that bypasses the natural defense mechanisms of the mucosal barrier of the upper respiratory tract, recent efforts have been invested in developing new standardized systems for natural and simulated natural FMDV exposure of cattle (19). Experimental investigations performed using aerosol (9, 14, 16) or intranasopharyngeal (10) inoculation of cattle have provided explicit, yet slightly differing details of the early events of FMDV pathogenesis.

Several investigations have used contact exposure to experimentally infect cattle with FMDV (19, 20); however, no previous study has performed detailed temporo-anatomic mapping of FMDV distribution subsequent to natural exposure. The objective of the current investigation was to further investigate the infection dynamics and tissue distribution of FMDV during early and late phases of infection following natural virus exposure. FMDV infection dynamics and temporo-anatomic distribution of virus in tissues were determined through standardized sample collection and tissue harvest at predetermined time points after virus exposure. This investigation provides detailed knowledge of FMDV pathogenesis under natural exposure conditions and serves to further evaluate commonly used experimental models for FMDV studies in cattle.

## RESULTS

### Animal experiments.

This study reports the outcome of FMDV infection in cattle after time-limited, direct contact exposure to FMDV-infected pigs. Six vaccinated and six nonvaccinated cattle were monitored and sampled through 35 days after contact exposure for the objective of investigating FMDV infection dynamics, as well as the occurrence and characteristics of FMDV persistence. Additionally, 12 nonvaccinated cattle were euthanized for tissue harvest during early infection (6 to 72 h postexposure [hpe]) for temporo-anatomic mapping of FMDV in bovine tissues during the acute phase of infection (Table 1).

**TABLE 1:** Cattle cohorts and numbers of cattle removed from rooms A to D at each predetermined time point

Room	Vaccination status	No. of animals euthanized at postexposure time point:					
		6 h	12 h	24 h	48 h	72 h	35 days
A	Nonvaccinated		2	2	2		
B	Nonvaccinated	2		2		2	
C	Nonvaccinated						6
D	Vaccinated						6

### Infection dynamics in cattle following exposure to FMDV-infected pigs. (i) Donor pigs.

**FIG 1:**
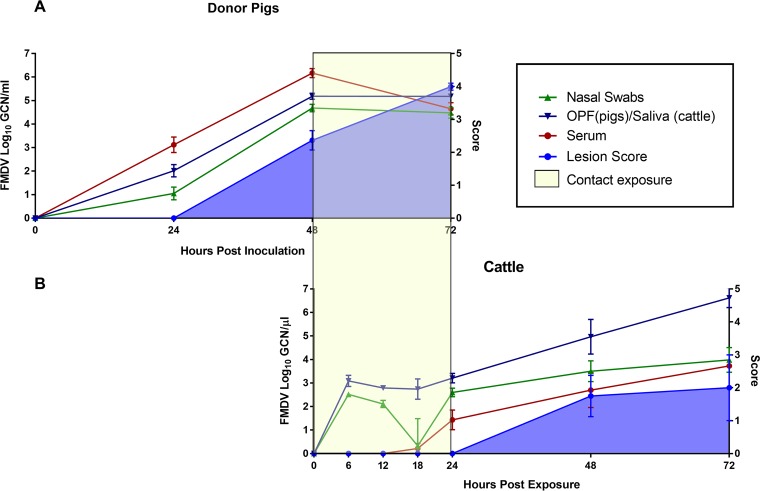
FMDV infection dynamics in pigs and cattle during early infection. (A) Detection of FMDV RNA by qRT-PCR in nasal swabs (green), oropharyngeal fluid (OPF; blue), and serum (red) from pigs (*n* = 12) from 0 to 72 h post-heel bulb inoculation. The blue-shaded area represents the cumulative lesion score. The FMDV-infected pigs were used as virus donors for cattle during a 24-h exposure period corresponding to 48 to 72 h postinoculation of the pigs (yellow box). (B) Detection of FMDV RNA by qRT-PCR in nasal swabs (green), saliva (blue), and serum (red) from cattle following 24 h of contact exposure to pigs (yellow box). The blue-shaded area represents the cumulative lesion score. There were 12 cattle at the start of the time course study, with 2 animals euthanized at each of 6, 12, 48, and 72 h postexposure (hpe) and 4 cattle euthanized at 24 hpe. The data represent geometric means ± SEM for all animals sampled at each time point.

The pigs that were used as virus donors during contact exposure were infected through heel bulb inoculation. The dynamics of infection were highly synchronous across all 12 pigs. All pigs were viremic, as determined by detection of FMDV RNA in serum, and were shedding detectable quantities of FMDV RNA in oropharyngeal and nasal secretions at 24 h postinoculation (hpi) (Fig. 1A). The first clinical signs of FMD, consisting of fever and vesicular lesions on coronary bands (noninoculated feet) and/or on the snout, were seen at 48 hpi in all pigs, which corresponded to the start of the cattle contact exposure. All pigs had severe generalized FMD at the end of the contact exposure period, at 72 hpi, at which time they were removed from the study and euthanized.

### (ii) Nonvaccinated cattle.

**FIG 2:**
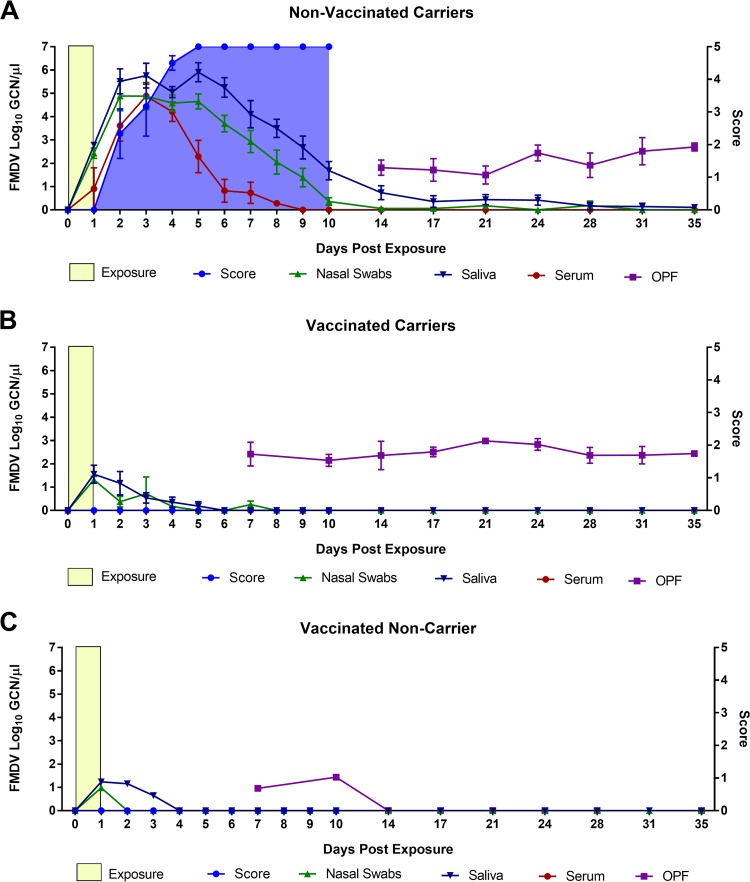
FMDV infection dynamics in cattle from early to persistent phases of infection. Shown is detection of FMDV RNA by qRT-PCR in nasal swabs, saliva, serum, and OPF from 0 to 35 days postexposure to FMDV-infected pigs (yellow boxes). The blue-shaded area represents the cumulative lesion score, which was recorded up to 10 days postexposure (dpe). There were no FMDV lesions in vaccinated cattle. FMDV carrier status was determined based on sustained detection of FMDV in oropharyngeal fluid (OPF [probang samples]). The graphs represent (A) nonvaccinated carriers (*n* = 6), (B) vaccinated carriers (*n* = 5), and (C) a vaccinated noncarrier (*n* = 1). The data points represent geometric means ± SEM for all animals sampled at each time point.

A total of 18 nonvaccinated cattle were included in the investigations (Table 1). Twelve of these were subjected to more intensive monitoring through the early phase of infection by sample collection at 6-h intervals through the first 24 h after the start of contact exposure. These 12 animals were euthanized for tissue harvest at predetermined time points between 6 and 72 h postexposure (hpe). The remaining 6 nonvaccinated cattle were sampled at 24-h intervals up to 10 days postexposure (dpe) and twice weekly through 35 dpe. Large quantities of FMDV RNA were measured in nasal and oral secretions from the nonvaccinated cattle as early as 6 h after the start of the contact exposure. This early detection of viral RNA in secretions may represent a combination of the environmental source (derived from the donor pigs), as well as replicating virus from newly infected cattle. Virus shedding in both oral and nasal secretions increased from 18 hpe (Fig. 1B) until peak levels were reached and were maintained through 2 to 5 dpe (Fig. 2A). The first detection of FMDV RNA in serum from cattle occurred at 18 hpe, and 10 out of 14 nonvaccinated cattle that were sampled at 24 hpe (corresponding to the end of contact exposure) were viremic at this time point (Fig. 1B and 2A). Serum levels of FMDV RNA declined from 4 dpe and were undetectable at 9 dpe (Fig. 2A). Seven out of 10 nonvaccinated cattle that were kept ≥48 hpe had vesicular lesions at this time point (Fig. 1B and 2A). There was a rapid progression of clinical FMD, and all cattle that were kept beyond the initial 72 hpe reached the maximum lesion score by 5 dpe, indicating that they had lesions in the mouth as well as on all four feet. Samples of oropharyngeal fluid (OPF [probang samples]) were collected from 14 to 35 dpe. All six nonvaccinated cattle that were kept through to the persistent phase of infection were confirmed to be FMDV carriers based on consistent detection of FMDV RNA and concurrent isolation of FMDV from OPF (Fig. 2A).

### (iii) Vaccinated cattle.

Six vaccinated cattle were included in the investigation for monitoring of FMDV infection dynamics and persistence following natural virus exposure. None of these cattle developed any clinical signs of FMD, and there was no detection of FMDV RNA in serum. However, all six cattle were subclinically infected, based upon detection of FMDV in secretions and/or tissues. Declining levels of FMDV RNA were detected in oral and nasal secretions in all animals from the end of contact exposure at 1 dpe until approximately 4 to 6 dpe (Fig. 2B and C). Similarly, FMDV RNA and infectious virus were recovered from OPF of all cattle at 7 and 10 dpe (Fig. 2B and C). One animal subsequently cleared infection (Fig. 2C), whereas 5 were persistently infected carriers, as determined by consistent detection of FMDV in OPF until the final sampling at 35 dpe (Fig. 2B).

### Tissue distribution of FMDV during early infection.

Twelve nonvaccinated cattle were euthanized during the early phase of infection (6 to 72 hpe) for determination of the temporo-anatomic progression of infection following natural virus exposure. The most consistent detection of FMDV in tissues prior to the onset of viremia occurred in the nasopharyngeal tissues. Specifically, infectious FMDV was isolated from the dorsal soft palate and/or the dorsal nasopharynx of all four cattle that were euthanized at 6 to 12 hpe (Table 1). Additional isolation of virus at these early time points occurred from the tongue, lingual tonsil, ventral soft palate, and submandibular lymph node (LN). One animal (ID 16-20) had slightly more extensive detection, with low to moderate quantities of FMDV RNA detected in the dorsal soft palate and dorsal nasopharynx concurrent with virus isolation (Table 2). Thus, the nasopharyngeal mucosa, including the dorsal nasopharynx and the dorsal surface of the soft palate, was the only site at which measureable quantities of virus were detected prior to dissemination of infection and onset of viremia. Small quantities of FMDV RNA and/or infectious virus were detected in the coronary band or interdigital cleft epithelium in three or the four previremic animals. This was interpreted as environmental contamination as these animals had been euthanized directly after removal from the room housing the infected pigs, and similar detection did not occur at subsequent time points.

**TABLE 2:** Tissue distribution of FMDV in nonvaccinated cattle during early infection

Parameter or tissue sample	Result at time point for animal with indicated ID number^*a*^:											
	Previremic/preclinical				Viremic/preclinical				Viremic/clinical			
	6 hpe		12 hpe		24 hpe				48 hpe		72 hpe	
	16-25	16-26	16-19	16-20	16-21	16-22	16-27	16-28	16-23	16-24	16-29	16-30
Clinical score	0	0	0	0	0	0	0	0	2	3	3	1
FMDV RNA copies/µl in serum	−	−	−	−	0.10	1.91	2.47	2.53	2.05	3.84	3.62	3.84
Tissue sample												
Oral cavity/oropharynx												
Tongue	+	−	−	−	+	1.88	1.57	3.82	+	8.83	7.87	6.61
Lingual tonsil	−	−	−	+	3.07	2.31	2.74	2.13	4.84	3.55	4.04	3.63
Palatine tonsil	−	−	−	−	3.33	3.07	4.95	+	4.65	5.71	5.27	3.73
Ventral soft palate	+	−	−	+	2.28	+	2.70	3.16	4.90	2.78	3.11	3.15
Nasopharynx												
Dorsal soft palate—rostral	−	−	−	4.46	2.72	3.22	3.82	3.48	4.07	3.53	4.06	3.63
Dorsal soft palate—caudal	+	+	+	1.66	5.30	4.12	4.64	4.16	4.68	3.57	3.24	4.53
Dorsal nasopharynx—rostral	+	+	−	2.70	5.48	3.70	4.27	6.14	3.91	2.88	3.51	3.62
Dorsal nasopharynx—caudal	−	−	−	2.57	4.80	3.71	4.02	2.77	4.60	2.77	3.52	3.62
Lateral nasopharynx	−	−	−	+	+	1.94	2.66	+	3.03	1.83	1.89	1.55
Nasopharyngeal tonsil	−	−	−	−	−	−	−	−	+	+	+	2.48
Lungs												
Proximal cranial lobe	−	−	−	−	−	+	2.37	+	+	2.60	3.58	3.04
Middle cranial lobe	−	−	−	−	−	+	+	2.31	+	2.97	4.04	2.27
Distal cranial lobe	−	−	−	−	−	+	+	+	+	3.01	4.01	2.16
Proximal middle lobe	−	−	−	−	+	+	2.55	+	+	2.58	2.85	2.68
Middle middle lobe	−	−	−	−	−	+	2.20	+	1.86	3.34	2.94	3.31
Distal middle lobe	−	−	−	−	−	+	1.97	+	−	2.73	4.43	3.89
Additional tissues												
Medial retropharyngeal LN	−	−	−	−	+	+	+	+	+	+	2.51	+
Submandibular LN	−	−	+	−	−	+	3.66	+	2.07	+	2.26	2.15
Hilar LN	−	−	−	−	−	+	+	+	+	2.82	+	+
Popliteal LN	−	−	−	−	−	+	+	+	2.17	3.92	2.85	+
Interdigital cleft	1.55	+	1.62	−	−	−	−	+	+	7.68	8.05	2.40
Coronary band	2.09	1.75	1.59	−	−	−	−	+	+	8.31	3.20	5.79

a Except as indicated, the numbers shown are GCN/mg (tissues) or GCN/µl (serum). Underlined numbers are values for samples positive by both qRT-PCR and virus isolation. Numbers not underlined indicate FMDV RNA detection without virus isolation. −, samples negative by both qRT-PCR and virus isolation; +, samples positive by virus isolation but negative by qRT-PCR. The clinical score represents FMDV lesion distribution, with a maximum score of 5 corresponding to lesions detected in the mouth and on all four feet.

All four cattle that were euthanized at 24 hpe were viremic, but had not yet developed any clinical signs of FMD. In these animals, moderate to large quantities of FMDV RNA were consistently detected in the nasopharyngeal tissues, concurrent with isolation of virus. Smaller quantities of FMDV RNA were detected in the tongue and oropharyngeal epithelium and oropharyngeal tonsils (Table 2). Infectious virus was isolated from lung tissues from all four animals, with concurrent detection of low levels of FMDV RNA in two out of the four cattle. The FMDV RNA quantities in the lungs during this early phase of infection were smaller than those in the nasopharynx and generally smaller than the quantities detected in serum.

The four cattle that were euthanized at 48 and 72 hpe were viremic and had characteristic vesicular lesion in the oral cavity and/or on one or two feet. During this more advanced stage of infection, FMDV was isolated from all sampled tissues (Table 2), interpreted as being consistent with detection of virus within the intravascular space. The highest viral loads were detected in vesicular epithelium from the tongue or feet. Three out of the four animals had consistent detection of FMDV RNA in all lung samples, whereas only one lung sample was positive by quantitative real-time reverse transcription-PCR (qRT-PCR) in the fourth animal (ID 16-23) (Table 2).

### Tissue distribution of FMDV during persistent infection.

All 6 nonvaccinated cattle and 5 out of 6 vaccinated cattle that were monitored through to the persistent phase of infection were FMDV carriers. Infectious virus and FMDV RNA were consistently detected in nasopharyngeal tissues (dorsal soft palate and dorsal nasopharynx) from the persistently infected carriers, regardless of vaccination status (Table 3). One nasopharyngeal sample harvested from the one animal that had cleared infection (ID 16-08) contained a small quantity of FMDV RNA, but without concurrent isolation of infectious virus, suggesting remnants of previous infection at this site. Similarly, small quantities of FMDV genome were detected in peripheral lymphoid tissue (submandibular and popliteal lymph nodes and palatine tonsils) from the nonvaccinated cohort, which is consistent with resolution of previously disseminated infection. Additionally, small quantities of FMDV RNA were detected in one lung sample and the associated hilar lymph node of one of the vaccinated carriers (ID 16-07), without isolation of infectious virus (Table 3).

**TABLE 3:** Tissue distribution of FMDV in vaccinated and nonvaccinated cattle during persistent infection

Parameter or tissue sample	Result for animal with indicated ID number^*a*^:											
	Nonvaccinated carriers						Vaccinated carriers					Vaccinated noncarrier (16-08)
	16-01	16-02	16-03	16-04	16-05	16-06	16-07	16-09	16-10	16-11	16-12	
FMDV RNA copies/ml in OPF	5.47	5.62	6.14	5.76	5.04	6.14	5.04	5.69	5.46	5.70	5.32	−
Tissue sample												
Oral cavity/oropharynx												
Tongue	−	−	−	−	−	−	−	−	−	−	−	−
Lingual tonsil	−	−	−	−	−	−	−	−	−	−	−	−
Palatine tonsil	−	3.15	2.02	−	2.19		−	−	−	1.87	−	−
Ventral soft palate	−	−	−	−	−	−	−	−	−	−	−	−
Nasopharynx												
Dorsal soft palate—rostral	3.25	3.02	4.38	4.14	3.27	5.46	3.73	4.10	3.16		4.84	−
Dorsal soft palate—caudal	2.70	3.42	5.13	4.76	5.29	5.29	5.22	2.04	4.35	4.38	3.71	1.85
Dorsal nasopharynx—rostral	4.59	4.36	−	4.37	3.25	5.73	4.36	2.41	3.36	3.82	4.34	−
Dorsal nasopharynx—caudal	3.00	3.09	4.14	3.43	−	2.30	5.12	−	2.51	−	2.44	−
Lateral nasopharynx—rostral	−	1.46	−	−	−	−	2.02	−	−	−	−	−
Lateral nasopharynx—caudal	−	2.62	−	3.33	1.72	−	2.31	−	−	−	−	−
Nasopharyngeal tonsil	−	−	−	3.27	3.38	−	−	−	−	−	−	−
Lungs												
Middle middle lobe	−	−	−	−	−	−	−	−	−	−	−	−
Distal middle lobe	−	−	−	−	−	−	2.22	−	−	−	−	−
Additional tissues												
Medial retropharyngeal LN	−	−	−	−	−	−	−	−	−	−	−	−
Submandibular LN	−	−	2.60	2.33	2.06	3.57	−	−	−	−	−	−
Hilar LN	−	−	−	−	−	−	2.50	−	−	−	−	−
Popliteal LN	2.51	2.55	1.96	−	2.05	−	−	−	−	−	−	−
Interdigital cleft	−	−	−	−	−	−	−	−	−	−	−	−

a Except as indicated, the numbers are GCN/mg (tissues) or GCN/ml (OPF). Underlined numbers are values for samples positive by both qRT-PCR and virus isolation. Numbers not underlined indicate FMDV RNA detection without virus isolation. −, samples negative by both qRT-PCR and virus isolation.

### Immunomicroscopy.

**FIG 3:**
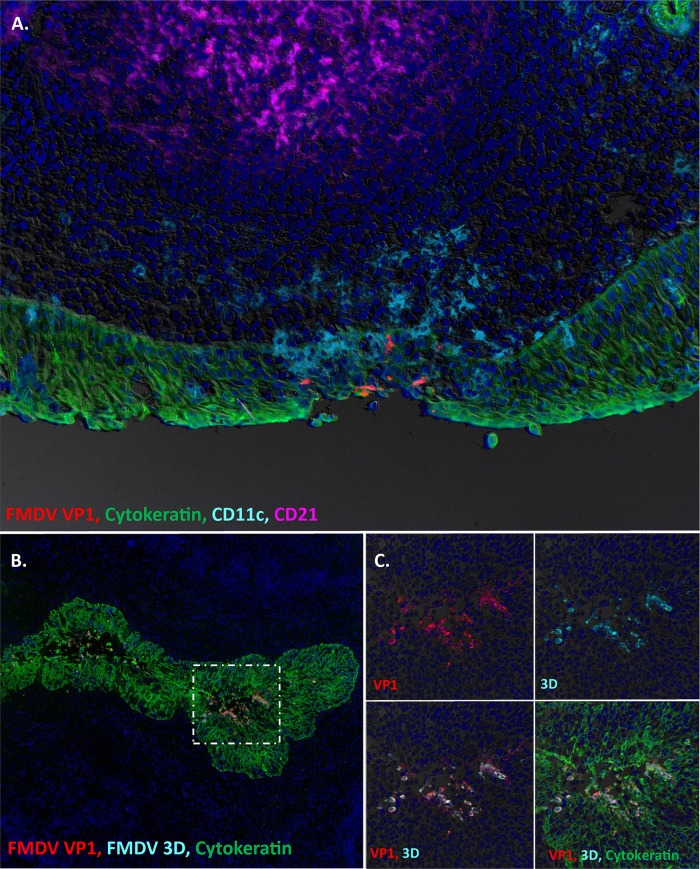
FMDV infection of nasopharyngeal mucosa and palatine tonsils of cattle during early stages of disease. (A) Primary FMDV infection of the dorsal nasopharyngeal mucosa of cattle at 24 h postexposure (hpe [animal ID 16-22]). FMDV VP1 (red) is localized to a focal area of MALT-associated epithelium of the dorsal nasopharynx, overlying a lymphoid follicle (purple). There is a partial-thickness erosion of the surface epithelium in association with the FMDV-infected area and a marked presence of CD11c^+^, presumed dendritic cells (turquoise). However, FMDV-infected cells are exclusively cytokeratin-positive epithelial cells (green). Magnification, ×20. (B) FMDV replication in an epithelial crypt of the bovine palatine tonsil at 48 hpe (animal ID 16-23). During this clinical stage of infection, large quantities of FMDV VP1 (red) and 3D (turquoise) protein are present within cytokeratin-positive (green) epithelial cells within the tonsil crypt. Magnification, ×10. (C) Higher-magnification and individual channel views of the area of interest boxed in panel B. Magnification, ×40.

Primary infection of the nasopharyngeal mucosa was confirmed by detection of FMDV VP1 (Fig. 3A) and 3D (not shown) by immunomicroscopy in tissue samples obtained at 24 hpe. Focal areas of infection were localized within distinct regions of lymphoid-associated epithelium and were associated with subtle erosions of the epithelial surface (Fig. 3A). Infiltration of CD11c^+^ cells (presumptive antigen-presenting cells) was seen in close proximity to FMDV-infected areas. At 48 to 72 hpe, substantial FMDV replication was detected in epithelial crypts of the palatine tonsils (Fig. 3B and C). At this more advanced stage of infection, large quantities of structural and nonstructural FMDV antigen were localized to focal regions of tonsillar crypt epithelium and were associated with acantholytic degeneration of the epithelium and the occurrence of intraepithelial microvesicular lesions (Fig. 3B and C). There was no detection of FMDV antigen in any pulmonary tissue samples harvested at any time of infection.

**FIG 4:**
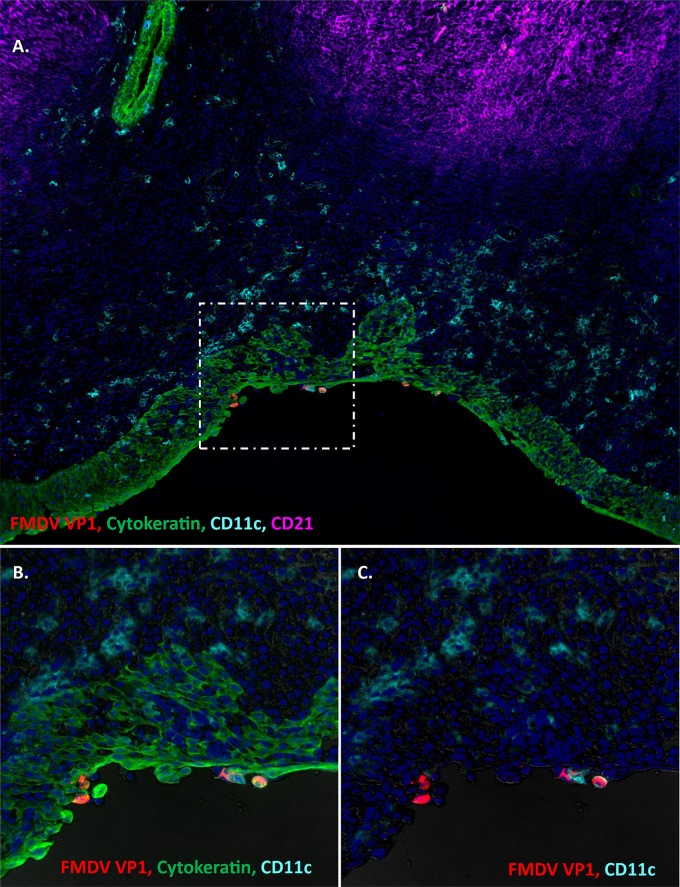
Persistent FMDV infection in the bovine nasopharyngeal mucosa. (A) FMDV infection in the dorsal nasopharyngeal mucosa at 35 days postexposure (animal ID 16-04). FMDV VP1 (red) is localized to cytokeratin-positive epithelial cells (green) within a segment of MALT-associated epithelium overlying a subepithelial lymphoid follicle of CD21^+^ B cells (purple). CD11c^+^ presumptive dendritic cells are abundant throughout the submucosa and interspersed within the epithelium, rarely colocalizing with virus-infected cells (C). However, there is no structural disruption of the tissue, and no evidence of a marked inflammatory reaction. Magnification, ×10. (B and C) Higher-magnification and individual channel views of the area of interest boxed in panel A. Magnification, ×40.

During the persistent phase of infection, the microscopic localization of FMDV VP1 (Fig. 4A) and 3D (not shown) was, similar to that in early stages of infection, localized to lymphoid-associated epithelium of the nasopharyngeal mucosa. Although the microanatomic localizations of virus during early and late stages of infection were similar, persistent FMDV infection was not associated with any structural alterations of the tissue or any evidence of a local inflammatory reaction. Although CD11c^+^ cells were abundant within the submucosa, there was no consistent clustering of these antigen-presenting cells associated with FMDV-infected foci (Fig. 4A). However, rare FMDV VP1-CD11c colocalization was detected within the epithelium.

### Detection of FMDV in air samples.

**FIG 5:**
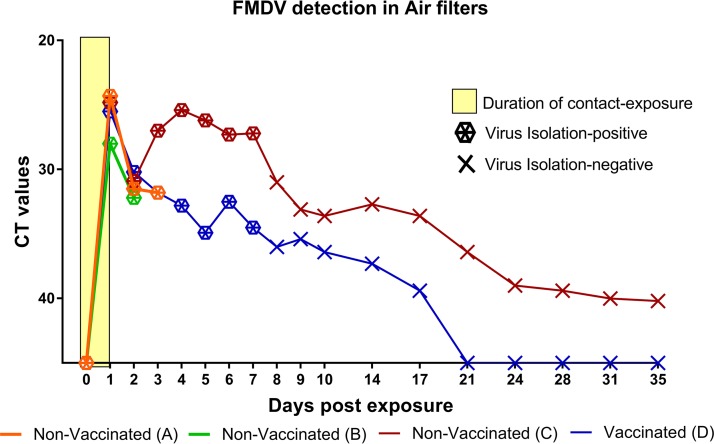
Detection of FMDV in air filter samples after exposure of cattle to infected pigs. Shown are the FMDV RNA quantities and isolation of infectious FMDV from dry air filters collected from isolation rooms housing vaccinated or nonvaccinated cattle that were exposed to FMDV-infected pigs for 24 h. The *y* axis represents FMDV RNA quantities (*C_T_* values) determined by qRT-PCR. Hexagon symbols indicate samples from which infectious virus was isolated, while X indicates virus isolation-negative samples. Rooms A and B housed nonvaccinated cattle (6 per room) that were euthanized at predetermined time points from 6 to 72 h postexposure. Rooms C and D housed nonvaccinated and vaccinated cattle, respectively (6 cattle per room), which were monitored through 35 days. Each room housed 3 FMDV-infected pigs from 0 to 1 day postexposure (yellow box). All 6 nonvaccinated cattle in room C developed clinical FMD, while none of the 6 vaccinated cattle in room D developed any signs of FMD.

Comparable amounts of FMDV RNA were detected in air samples in all 4 rooms at the end of the contact exposure (1 dpe). Similarly, viral RNA quantities in air samples declined in all rooms during the first 24 h after removal of the infected pigs from the rooms (2 dpe) (Fig. 5). From 2 to 3 dpe, detected FMDV RNA quantities increased in room C, which housed 6 nonvaccinated cattle, but continued to decrease in room D, housing 6 vaccinated cattle. The quantities of viral RNA detected in room C, with nonvaccinated cattle, remained at similarly high levels through to 7 dpe, after which these started to decline. Viral RNA remained detectable in air samples from the room with nonvaccinated cattle through the experimental period, but decreased below detectable limits by 21 dpe in the room with vaccinated animals. Infectious FMDV was isolated from the air samples from all 4 rooms through the early phase of infection and up to 7 dpe in rooms C and D, in which sampling was continued up to 35 dpe (Fig. 5).

### FMDV full-coding-sequence analysis.

**FIG 6:**
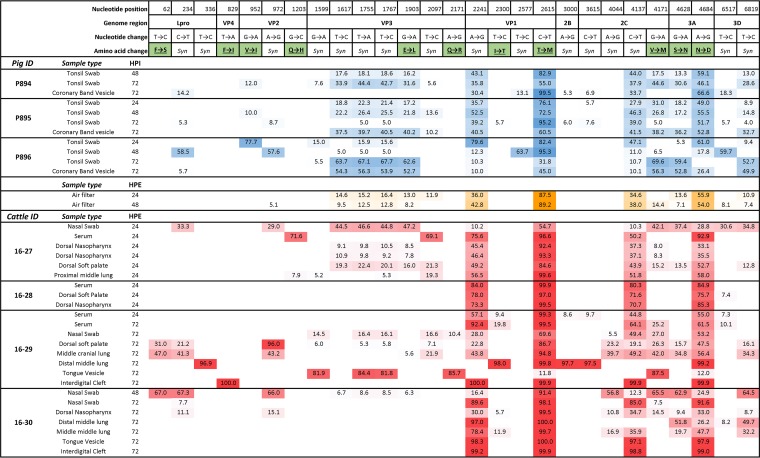
Subconsensus single nucleotide polymorphism (SNP) frequencies in porcine and bovine FMDV samples during early infection. Shown are frequencies (percentages) of SNPs, compared to the inoculum consensus sequence, present at ≥5% in samples obtained from 3 pigs and 4 cattle that were housed in room A. All animals were housed together for 24 h, corresponding to 48 to 72 h postinoculation (hpi) of the pigs and 0 to 24 h postexposure (hpe) of the cattle. Cattle 16-27 and 16-28 were euthanized for tissue harvest at 24 hpe, and cattle 16-29 and 16-30 were euthanized at 72 hpe. Additionally, FMDV sequence was obtained from air samples in the room at 24 and 48 hpe. The numbers in the figure represent the percentages of distinct SNPs in each sample. Color gradients indicate increasing frequency of SNP detection with greater color intensity. Blue, porcine samples; yellow, air samples; red, bovine samples. *Syn*, synonymous nucleotide change.

Samples obtained from cattle and pigs in room A were subjected to FMDV deep sequence analysis by next-generation sequencing (NGS). This experimental cohort included 3 pigs that were all euthanized at 72 h postinoculation (hpi), as well as 6 cattle, of which 2 were euthanized for tissue collection at each of 6, 24, and 72 hpe (cattle animal IDs 16-25 through 16-30) (Table 2 and Fig. 6).

It was not possible to identify definitive transmission chains based on FMDV consensus sequence phylogenetic analysis due to the high similarity between consensus sequences (maximum *p*-distance = 0.002; 13 nucleotides [not shown]). However, the frequency distribution of subconsensus viral variants provided insights into FMDV pathogenesis events.

The original inoculum used to infect the pigs contained a highly diverse viral population. Consequently, during early infection, viral populations in pig-derived samples had abundant synonymous and nonsynonymous single nucleotide polymorphisms (SNPs) compared to the inoculum consensus sequence (Fig. 6). A single nonsynonymous SNP in VP2 (nucleotide [nt] position 952) was detected at low to high frequency in at least one sample from all pigs, but was absent from all cattle samples. Interestingly, four VP3 nucleotide variants (nt positions 1617, 1755, 1767, and 1903) were common in almost all pig samples. The only other samples to contain all of these four variants were two air samples, and nasal swab and upper respiratory tract samples from a single cow (ID 16-27). Of these four VP3 SNPs, the nucleotide change at position 1903 was the only nonsynonymous change (E to L). Four nonsynonymous changes occurred in cattle samples only (nt positions 62, 829, 1203, and 2171). These SNPs were found in a total of eight samples from three different animals and were dominant, reaching frequencies of 67 to 100, in four of those samples. Two nonsynonymous substitutions—threonine to methionine at VP1^147^ (end of the G-H loop) and asparagine to aspartate at 3A^136^—were detected at the consensus level in at least one sample from all 3 donor pigs and were also present at high frequencies in all sampled cattle. Of the remaining synonymous changes, three (nt positions 2241, 4137, and 6517) occurred in at least one sample from every animal in the study.

Vesicular lesions obtained from cattle during the clinical phase of disease (72 hpe) contained viral populations that were substantially less diverse (nearly clonal) than samples obtained from nasopharyngeal tissues at corresponding time points. Pulmonary tissue samples contained virus populations of mixed characteristics: these were either highly diverse, and thus, similar to nasopharyngeal tissues (e.g., animal 16-29, middle cranial lung [Fig. 6]), or close to clonal, thereby more resembling vesicular lesions (e.g., animal 16-29, distal middle lung [Fig. 6]). Strikingly, samples consisting of close to clonal but distinctly different populations were obtained from different anatomic sites at the same time point of one animal (animal 16-29, interdigital cleft vesicle, distal middle lung at 72 hpe [Fig. 6]).

## DISCUSSION

Multiple previous works have investigated FMDV pathogenesis in cattle. However, differences in study design, specifically with regard to methods used for virus exposure, have led to various findings. The objective of the current investigation was to utilize a natural but controlled system of direct contact exposure to achieve a detailed understanding of the progression of FMDV infection in cattle, from primary infection through systemic generalization to establishment of persistent infection.

The experimental model included inoculation of donor pigs with a cattle-derived FMDV strain. Infected pigs were subsequently moved into clean rooms housing naive or vaccinated recipient cattle, at a time point that corresponded to the early clinical phase of FMD in the pigs. Initiating the contact exposure at a time at which the pigs were known to be shedding large quantities of FMDV (21) and limiting the exposure to 24 h provided a relatively uniform exposure of the cattle and also enabled standardization of the contact exposure. Thus, the currently used experimental system provided a standardized and controlled model for natural FMDV exposure of cattle that is suitable for detailed time course pathogenesis studies.

The onset and progression of FMD in exposed cattle were rapid and consistent. The first detection of viremia in nonvaccinated cattle occurred as early as 18 h after the start of contact exposure. Similarly, the majority of nonvaccinated cattle were viremic at 24 hpe, and all had vesicular lesions in the mouth or on at least one foot at 48 hpe. This very rapid disease progression is similar to that which occurs after conventional tongue inoculation of cattle with virulent FMDV strains (20, 22). Additionally, the within-group variation in infection dynamics was lower than previous studies in which the same virus strain was used to infect cattle by simulated natural intranasopharyngeal inoculation (10). This comparison to previous studies with the same virus suggests that the effective challenge dose received by cattle in the current investigation was both high and consistent across animals. The current finding of primary FMDV infection within the nasopharyngeal mucosa is consistent with previous investigations based on experimental aerosol or intranasopharyngeal inoculation (9, 10). However, in contrast to investigations based on aerosol inoculation (9), the low quantities of virus recovered from pulmonary tissues during previremic or early viremic stages of disease in the current study were not suggestive of substantial virus replication occurring in the lungs before the onset of viremia.

Similar to previous investigations, vaccination with an adenovirus-vectored FMD A vaccine 14 days prior to virus exposure conferred complete protection against clinical FMD and viremia (10, 12). All vaccinated animals were, however, subclinically infected, as determined by detection of FMDV in oropharyngeal secretions and in tissues and 11 out of 12 cattle (6 nonvaccinated and 5 vaccinated) that were kept through the persistent phase of infection and were confirmed to be FMDV carriers.

Analysis of tissue samples from early and persistent stages of disease by immunomicroscopy confirmed localization of both primary and persistent FMDV infection to specialized regions of epithelium of the nasopharyngeal mucosa overlying the mucosa-associated lymphoid tissue (MALT). This finding confirms that the simulated natural, aerosol (9, 23), and intranasopharyngeal (INP) (10, 12) challenge systems result in similar pathogenesis in cattle, as was demonstrated herein by natural contact exposure.

Despite the identical microanatomic locations of virus associated with primary versus persistent FMDV infection, there were notable differences in histopathological characteristics. Consistent with previous investigations (9, 10), primary FMDV infection of the bovine nasopharynx was associated with erosions of the mucosal surface, as well as marked infiltration of antigen-presenting cells concurrent with establishment of viremia. Additionally, the more advanced stage of early infection was associated with substantial virus replication occurring in palatine tonsil crypt epithelium, with marked acantholytic degeneration of the epithelium in infected foci. This morphologic feature in the tonsil epithelium is essentially the same process that occurs in FMD vesicles at peripheral lesion sites (24). In contrast to this, there was no evidence of an activated host response associated with persistent FMDV infection of the nasopharyngeal mucosa. During the carrier state, FMDV-infected single epithelial cells or small clusters of epithelial cells were dispersed within similar regions of MALT epithelium, but without associated structural damage or local inflammatory activation. These findings are consistent with previous studies which have suggested inhibition of the host antiviral response, as well as induction of immunological tolerance in association with FMDV persistence (12, 25, 26).

Viral RNA quantities detected in air samples in room C, with 6 nonvaccinated cattle, increased from 2 to 3 dpe, after removal of the pigs, suggesting that the infected cattle contributed to airborne virus during the clinical phase of disease. Contrastingly, the quantities of viral RNA detected in room D, with 6 vaccinated cattle, decreased continuously after removal of the pigs from the room. Despite this, infectious FMDV was isolated as late as 7 dpe in filters from both rooms. The combination of sequence similarity between pig samples and air samples and previous observations confirming that in a similar setting, vaccinated and protected cattle did not shed detectable virus into the air (unpublished data), suggests that the airborne virus in the room of the vaccinated cattle may to a large extent have been derived from the pigs.

Analysis of the frequency of FMDV genome substitutions below and above the consensus level provided interesting insights into the complexities of FMDV pathogenesis during early infection. This part of the investigation included only a limited subset of study animals and was limited to unpassaged sample material to avoid introduction of artifacts occurring during viral amplification in cell culture. Overall, there was a consistent trend that viruses obtained from vesicular lesions of cattle were highly homogenous, approaching clonality, whereas the viruses obtained from nasal swabs, air, and most tissues had highly heterogenous virus populations (swarms). This finding is consistent with previous reports that concluded that FMDV samples from vesicular epithelium were less diverse than esophagopharyngeal scrapings, suggesting within-host bottleneck events in association with formation of FMDV vesicles (27, 28). Herein, we additionally demonstrated the simultaneous detection of highly homogeneous yet distinct clones from different anatomic sites of the same animal, suggesting that multiple independent bottleneck events occurred within individual animals. This simultaneous occurrence of virus populations of distinct genetic patterns and different degrees of diversity within an infected host may have implications for onward transmission of virus. However, it is unclear whether transmission is more likely to initiate from vesicular lesions, with very high quantities of virus with a narrow population structure, or if the greater diversity of the virus populations found in oral and nasal secretions results in an increased likelihood of transmission despite smaller quantities of virus.

The virus samples obtained from the inoculated pigs were highly diverse, reflecting the high diversity found in the original virus inoculum. In contrast to cattle-derived samples, a similar level of diversity was seen in samples from the site of primary virus replication (tonsil swabs), as in vesicular epithelium (coronary band lesions) in the pigs. This suggests a lesser extent of bottleneck events within the pigs compared to the more extensive effects that occurred as part of the process of transmission, adaptation, and pathogenesis within a new host species. Additionally, the species-specific virus populations may represent differential species-specific viral fitness defined by host selective pressures.

The different levels of within-animal virus diversity across the different cattle may be suggestive of different mechanisms of virus transmission or the occurrence of bottlenecks associated with transmission from one host to another. For example, the striking homogeneity of virus samples obtained from animal 16-28 suggests that infection in this animal was likely founded by a narrow population. In contrast to this, the greater diversity of viruses in animals 16-27 and 16-29 suggests that these animals were likely exposed to different or more diverse founding virus populations. The highly synchronous infection dynamics across the cattle suggests that all animals received a comparable challenge dose. However, the differences in the diversity of within-host virus populations suggest that there may still have been qualitative differences in how these animals acquired the primary infection.

### Conclusion.

The primary output of this investigation was confirmation of the importance of the bovine nasopharyngeal mucosa as the site of both primary and persistent FMDV infection after natural contact exposure. This confirms the output of many previous studies that reached similar conclusions using simulations of natural infection. Additionally, natural virus exposure did not cause substantial FMDV replication in the lower respiratory tract prior to establishment of viremia. The standardized experimental system of time-limited exposure to FMDV-infected pigs provided a stringent and consistent virus challenge across different cohorts of animals, providing a useful tool for FMDV pathogenesis studies. The output of the FMDV sequence analyses demonstrated that FMDV quasispecies compositions differ substantially between primary and secondary replication sites. The current findings provide novel insights on the within- and between-host evolution of FMDV, which are being explored in greater detail in ongoing investigations within our laboratory.

## MATERIALS AND METHODS

### Virus.

The virus used for this investigation was a cattle-derived strain of FMDV A_24_ Cruzeiro that has been characterized in detail in previous pathogenesis studies (10, 12, 29).

### Vaccine.

The six cattle in room D were vaccinated 14 days prior to virus challenge by intramuscular injection of an adenovirus-vectored FMDV serotype A vaccine as previously described (12, 30, 31).

### Animal experiments.

Animal experiments were performed within biosafety level 3 agriculture (BSL3Ag) biocontainment facilities at the Plum Island Animal Disease Center, New York. All procedures were performed in compliance with an experimental protocol approved by the Institutional Animal Care and Use Committee (protocol 231.03-14-R). Cattle were Holstein heifers weighing approximately 150 to 200 kg when delivered. Pigs were castrated Yorkshire males weighing approximately 25 to 30 kg. Pigs and cattle were housed in separate isolation rooms before virus challenge.

### (i) FMDV challenge.

The study included a total of 24 cattle and 12 pigs. Pigs were inoculated through intraepithelial injection in the heel bulb of one foot as previously described (32). Each pig received a dose of 10^5^ BTID_50_ (50% infectious doses titrated in bovine tongue epithelium), diluted in minimal essential medium (MEM) to a total volume of 0.4 ml. At 48 h postinoculation (hpi) of the pigs, the pigs were moved into different isolation rooms that housed the cattle. After 24 h of cohabitation, the pigs were removed from the rooms and euthanized. During each contact exposure trial, 3 pigs were housed together with 6 cattle in a room with an available floor area of 27 m^2^, a total air volume of approximately 135 m^3^, and 28 complete air exchanges per hour. The study included replicate challenge trials performed in four separate rooms. Rooms A and B housed nonvaccinated cattle that were included in investigations of FMDV pathogenesis during early stages of infection. These animals were euthanized at predetermined time points between 6 and 72 h postexposure (hpe). Cattle in rooms C and D were kept for 35 days for the objective of investigating FMDV persistence. Among these animals, there were 6 vaccinated and 6 nonvaccinated individuals. During contact exposure of cattle in rooms C and D, 3 vaccinated and 3 naive cattle were housed together with 3 donor pigs to ensure uniform exposure conditions for all cattle. Vaccinated and nonvaccinated cattle were subsequently separated and moved into different rooms after 24 h of cohabitation with the infected pigs: nonvaccinated in room C and vaccinated in room D.

### (ii) Sample collection.

FMDV infection dynamics and virus shedding in pigs were monitored through collection of blood, nasal, and oropharyngeal (OP) swabs at 24-h intervals from 0 to 72 hpi. Swabs from pigs were immersed in 2 ml of MEM with 25 mM HEPES. The progression of clinical FMD in pigs was recorded using a cumulative scoring system as previously described (32, 33). Samples from cattle consisted of blood, oral, and nasal swabs. During the trials investigating early pathogenesis (rooms A and B), samples were collected at 6-h intervals from 0 to 24 h postexposure (hpe) and at 24-h intervals subsequently. For investigations of FMDV persistence, samples were collected once daily from 0 to 10 days postexposure (dpe). From 10 to 35 dpe, blood samples were collected once per week, and swabs were collected twice weekly. Additionally, oropharyngeal fluid (OPF) samples were collected twice weekly by use of a probang cup (34), starting at 14 dpe in nonvaccinated cattle and 7 dpe in vaccinated cattle. OPF samples were diluted with an equal volume of MEM containing 25 mM HEPES and were homogenized using a 16-G cannula attached to a 30-ml syringe. One OPF aliquot intended for virus isolation was treated with 1,1,2-trichlorotrifluoroethane (TTE) for dissociation of immune complexes prior to freezing (35, 36). Blood and swabs were centrifuged for harvest of serum or secretions prior to freezing. All sample aliquots were frozen at −70°C until further processing. The progression of clinical FMD in cattle was determined using a cumulative score for which any lesion in or around the oral cavity contributed 1 point and vesicular lesions on the feet contributed another point per foot, giving a maximum score of 5. Cattle were sedated by intramuscular injection of xylazine (0.66 mg/kg of body weight) to enable clinical examination of the feet. Sedation was reversed by intravenous injection of tolazoline (2.0 mg/kg of body weight). Naïve cattle were sedated once daily until reaching a full lesion score, which in all animals occurred between 3 and 5 dpe. Vaccinated cattle were sedated for clinical examination at 0, 3, 7, and 10 dpe.

### (iii) Postmortem sample collection.

For the objective of evaluating tissue distribution of FMDV during early infection, two nonvaccinated cattle were euthanized for tissue harvest at 6, 12, 48, and 72 hpe, and four animals were euthanized at 24 hpe. For evaluation of tissue distribution during the persistent phase of infection, six vaccinated and six nonvaccinated cattle were euthanized at 35 dpe. A standardized necropsy procedure with collection of 18 to 22 distinct tissue samples (Tables 1 and 2) was performed immediately after euthanasia. Each tissue sample was divided into 30-mg aliquots, which were placed in individual tubes before immediately being frozen over liquid nitrogen. An adjacent specimen from each tissue was divided into two or four replicates, embedded in optimal cutting temperature medium (Sakura Finetek, Torrance, CA) in cryomolds, and frozen over liquid nitrogen. Tissue samples were kept frozen in the vapor phase over liquid nitrogen and were transferred to the lab within 2 h after collection for storage at −70°C until further processing.

### (iv) Air sampling.

Air sampling was performed using a model 1000 air pump developed by the Program Executive Office for Chemical Biological Defense (PEO-CBD), fitted with an original DFU filter assembly holding two separate Lockheed Martin polyester filter discs (1.0-μm-pore filter, diameter 47 mm; catalog no. DFU-P-24; Lockheed Martin, Washington, DC) as previously described (37). The airflow through the unit was 15 liters/min, and the pump was placed out of reach of the animals. The filter discs were removed and replaced at 24-h intervals. The pump was left running in between daily sample collections, and the filters were removed prior to cleaning of the animal rooms, with the pump turned off as the room was cleaned to avoid sampling of artificially created aerosols.

### FMDV RNA detection.

Two aliquots of each tissue sample collected at necropsy were thawed and individually macerated in tissue culture medium, using a TissueLyser bead beater (Qiagen, Valencia, CA) and stainless steel beads (Qiagen catalog no. 69989). Air filters were cut into quarters and disrupted using a similar approach as for tissue samples, but with washed glass beads (combination of bead sizes 425 to 600 µm and ≤106 µm; Sigma catalog no. G4949/G8772). The air filter “homogenate” was subsequently centrifuged to extract the fluid that had been absorbed by the filter. Total RNA was extracted from tissue macerates, air filter supernatants, serum, swabs, and OPF samples using Ambion’s MagMax-96 viral RNA isolation kit (Ambion, Austin, TX) on a King Fisher-96 magnetic particle processor (Thermo Scientific, Waltham, MA). Extracted RNA was analyzed using quantitative real-time reverse transcription PCR (qRT-PCR), targeting the 3D region of the FMDV genome (38) with forward and reverse primers adapted from Rasmussen et al. (39) and chemistry and cycling conditions as previously described (40). Cycle threshold (*C_T_*) values were converted to RNA copies per milliliter or milligram using an equation derived from analysis of serial 10-fold dilutions of *in vitro*-synthesized FMDV RNA of known concentration. The equations of the curve of RNA copy numbers versus *C_T_* values were further adjusted for average mass of tissue samples and specific dilutions used during processing of samples. The qRT-PCR results reported in Tables 1 and 2 are the higher FMDV genome copy number (GCN)/mg value of the 2 samples processed per tissue per animal. Results reported in Fig. 1 and 2 represent the geometric mean (±standard error of the mean [SEM]) log_10_ GCN/µl for all animals sampled at each time point. Results for analysis of air filters are reported as *C_T_* values.

### Virus isolation.

Aliquots of macerated tissue samples, air filter supernatants, and TTE-treated probang samples were cleared from debris and potential bacterial contamination by centrifugation through Spin-X filter columns (pore size of 0.45 μm; Sigma-Aldrich). Samples were subsequently analyzed for infectious FMDV through virus isolation (VI) on LFBK-αvβ6 cells (41, 42), following a protocol previously described (16). All VI cell culture supernatants were analyzed by qRT-PCR, as described above, to confirm presence or absence of amplified FMDV.

### Immunomicroscopy.

After screening of tissue samples for FMDV RNA and infectious virus by qRT-PCR and VI, respectively, detection of antigen in cryosections by immunohistochemistry (IHC) and multichannel immunofluorescence (MIF) was performed as previously described (9, 24). Slides were examined with a wide-field, epifluorescence microscope, and images were captured with a cooled, monochromatic digital camera. Images of individual detection channels were adjusted for contrast and brightness and merged in commercially available software (Adobe Photoshop CC). Alternate sections of analyzed tissues were included as isotype controls, and additional negative-control tissue sections were prepared from corresponding tissues derived from noninfected cattle. Nonstructural FMDV (3D) protein was detected using the mouse monoclonal antibody F19-6 (43), and FMDV structural protein (VP1) was detected using the mouse monoclonal antibody 6HC4 (44). MIF experiments included labeling of phenotypic cell markers using the following antibodies: rabbit anti-cytokeratin (180059; Life Technologies), mouse anti-CD21 (ab1090; Abcam), mouse anti-bovine CD11c (BOV2026; Washington State University), as well as isotype control antibodies for mouse IgG1 and IgG2b (MG100 and MG2B00; Invitrogen).

### FMDV sequence acquisition and analysis.

Samples obtained from cattle and pigs in room A were subjected to FMDV deep sequence analysis by Illumina-platform, next-generation sequencing (NGS). This experimental cohort included 3 pigs that were all euthanized at 72 hpi, as well as 6 cattle, of which 2 were euthanized for tissue collection at each of 6, 24, and 72 hpe (cattle animal IDs 16-25 through 16-30 [Tables 1 and 3]). Initial sample selection from pigs included tonsil swabs obtained at 24, 48, and 72 hpi, as well as coronary band vesicle epithelium harvested at 72 hpi. Antemortem cattle samples included nasal swabs and serum, and postmortem tissue samples that were selected based on virus distribution in individual animals, with the objective of including samples representing the primary infection site (nasopharynx), lungs, and secondary lesion sites (tongue or interdigital cleft) when possible. The sample set that was ultimately included in the NGS analysis was determined based on the ability to obtain overlapping amplicons (details below) corresponding to the FMDV open reading frame (ORF) from unpassaged samples. Overlapping amplicons were generated using FMDV A_24_-specific primers with the SuperScriptIII kit per the manufacturer’s instructions (Thermo Fisher Scientific). Amplicons were quantified using the Qubit 2.0 fluorometer, and NGS libraries were constructed using the Nextera XT DNA library kit (FC-131-1096; Illumina) and run on a NextSeq 500. Reads were quality trimmed and mapped to the inoculum consensus sequence using CLC Genomics Workbench v9.5 (Qiagen). Full ORF consensus sequences were subjected to a minimum coverage of 10 reads with quality scoring/vote used for ambiguous base calls. Consensus sequences were aligned using MUSCLE (45), implemented in MEGA7 (46), and a phylogenetic tree was constructed using a maximum likelihood, HKY+G nucleotide substitution model and 100 bootstrap replicates implemented in MEGA7. The tree was visualized in MEGA7, with the inoculum designated as the root. Additionally, sites of subconsensus variation from the inoculum consensus sequence were identified for individual samples. Only variation sites reaching fixation in at least one sample, with ≥5% frequency, with a ≥0.25 forward/reverse ratio, and having adequate coverage were evaluated.

### Data availability.

FMDV consensus sequence data are available at GenBank with the following sample identification numbers: inoculum, MH746921, and animal and air samples, MH559773 to MH559806. NGS data for animal and air samples are available at the Sequence Read Archive with sample identifiers SAMN09580658 to SAMN09580694.
